# Recent Advances in Rare Earth Element Recovery: Liquid–Liquid Extraction and Magnetophoretic Separation

**DOI:** 10.1021/acs.iecr.5c02137

**Published:** 2025-10-06

**Authors:** Bailey Lake, Theo Siegrist, Thomas E. Albrecht, Hadi Mohammadigoushki, Munir Humayun, Jamel Ali

**Affiliations:** † Department of Chemical and Biomedical Engineering, 86485 FAMU-FSU College of Engineering, Tallahassee, Florida 32310, United States; ‡ National High Magnetic Field Laboratory, Tallahassee, Florida 32310, United States; § Department of Chemistry and Nuclear Science and Engineering Center, 3557 Colorado School of Mines, Golden, Colorado 80401, United States; ∥ Center for Rare Earths, Critical Minerals, and Industrial Byproducts, National High Magnetic Field Laboratory, Tallahassee, Florida 32310, United States; ⊥ Department of Earth, Ocean and Atmospheric Science, 7823 Florida State University, Tallahassee, Florida 32304, United States

## Abstract

The unique physicochemical properties of rare earth elements (REEs) make them imperative for the production and improvement of advanced technology, renewable energy, and national defense applications. As these fields rapidly develop, there has been an increase in the demand for REEs. Due to the similar ionic charge and radii of adjacent REEs it is a challenge to selectively recover individual elements. Currently, the most common method for industrial REE separation is multistep traditional liquid–liquid extraction (T-LLE) systems. However, these systems generate large quantities of hazardous waste including flammable solvents, contaminated wastewater, and materials that cannot be recycled. There has been a recent increase in modifications to T-LLE systems, which has shown promising results in improving REE refinement and decreasing waste. This Review summarizes recent advances in solvent extraction systems other than T-LLE including aqueous two-phase systems (ATPS), nonaqueous systems (NAS) and systems evaluating the synergistic effects of multiple extractants or solvents in each phase. We also discuss developments in magnetophoretic separation methods that could be useful in solvent extraction in the future. Based on the findings of this Review, estimated Technology Readiness Levels (TRLs) for each of these systems are provided. These new systems are currently at TRLs of ≤5. Improving their TRLs and examining their industrial potential could lead to a reduction in operation cost and environmental impacts while improving separation efficiency and overall REE refinement, if practical in industrial settings.

## Graphical abstract

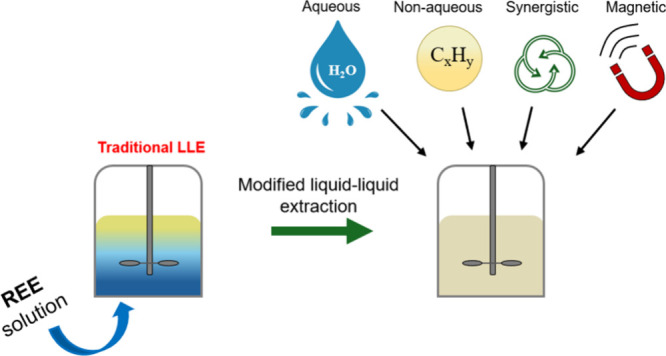

## 1 Introduction

The demand for rare earth elements (Sc, Y, and all 15 lanthanides) is increasing as there is a global effort toward improving clean technology and renewable energy applications. Rare earth elements (REEs) are considered energy critical elements (ECEs) as they are facing supply risks and are vital to the production of wind turbine generators, traction motors and batteries in electric vehicles, light-emitting diodes, and more (Table 1) which will inevitably see production increases in the foreseeable future.^1^ Progress in the development of rare earth doped inorganic materials is also expected to improve quantum information processing, telecommunication, and composite materials and structures used in predator drones, F-22 fighter jets, radar detection, and nuclear submarines.^2–4^ In recent years, many countries have reported REEs as critical materials and have put forth initiatives to focus on REE sourcing and production.^5–8^ REEs are recovered through mining and processing and through recycling secondary sources; although, limited quantities are actually being recycled.^9^ Geopolitical competition is involved with these critical materials, so sourcing and balancing the supply and demand is of major concern.^10^ Wang et al. proposes three strategies to address these issues: *diversifying supply*, *developing substitute materials*, and *urging reuse, recovery, and efficient use of materials in manufacturing*.^11^ Diversifying the supply includes more mining operations in the United States and a focus on improving REE refinement in the U.S. as there are approximately 1.5 million metric tons of total rare earth oxides (TREOs) in domestic reserves and inferred resources.^12^ This includes 1.12 million tons at Mountain Pass in California and 384000 tons at Bear Lodge in Wyoming. An additional estimated 10.3 million metric tons of TREOs are present in unclassified resources.^12^ However, the number of prospective new REE projects are small, so partnering with reliable trading partners (e.g., Australia and Canada) would not only diversify the REE supply but help meet production demands.^12^ Current REE sources from the U.S. are primarily being shipped to China for processing because of lower production costs due to environmental guidelines and resources. The U.S. is not alone in outsourcing REEs as sources mined in Mount Weld, Australia, are being shipped to Malaysia.^10^

**Table 1:** Rare-Earth Elements and Their Common Applications

element	elemental ions	atomic number	applications^10,14^
lanthanum	La(III)	57	Optics, batteries, catalysis, glasses, ceramics, pigments
cerium	Ce(III)	58	Chemical applications, coloring, catalysis, polishing powders, ceramics, UV filters
praseodymium	Pr(III)	59	Magnets, lighting, optics, ceramics, glasses, pigments
neodymium	Nd(III)	60	Magnets, lighting, lasers, optics, catalysts, IR filters
promethium	Pm(III)	61	Paint, miniature nuclear batteries; very rare in nature
samarium	Sm(III)	62	Magnets, lasers, masers, microwave filters
europium	Eu(III)	63	Lasers, lighting, medical applications
gadolinium	Gd(III)	64	Magnets, glasses, lasers, X-ray generation, computer applications, medical imaging, ceramics, glasses
terbium	Tb(III)	65	Lasers, lighting
dysprosium	Dy(III)	66	Magnets, lasers, ceramics
holmium	Ho(III)	67	Lasers, ceramics
erbium	Er(III)	68	Lasers, steelmaking, glasses, optical fibers
thulium	Tm(III)	69	X-ray generation, medical imaging
ytterbium	Yb(III)	70	Lasers, chemical industry applications, metallurgy
lutecium	Lu(III)	71	Medical applications, chemical industry applications, single crystal scintillators
scandium	Sc(III)	21	Alloys in aerospace engineering, lighting, electron bean tubes
yttrium	Y(III)	39	Lasers, superconductors, microwave filters, lighting, capacitors, glasses, sensors, radars

In the United States, REE production began in the early 1950s after Molybdenum Corporation of America (Molycorp) started extracting them from carbonatite ore in Mountain Pass, CA, and in the 1960s and 70s, production there increased after the rapidly growing demand of REEs due to the use of europium in colored television screens, samarium in permanent magnets, and gadolinium in X-ray shielding.^13^ It was not until 1992 that China surpassed the U.S. in REE production due to investments and supervision from both government and private sectors.^13^ Since then, U.S. domestic production has not been adequate for the growing demand of REEs. In 2023, $190 million worth of REE compounds and metals were imported into the U.S. with a net import reliance greater than 95%.^9^ Figure 1 shows the percentage of REE sources from each importing country.

**Figure 1:**
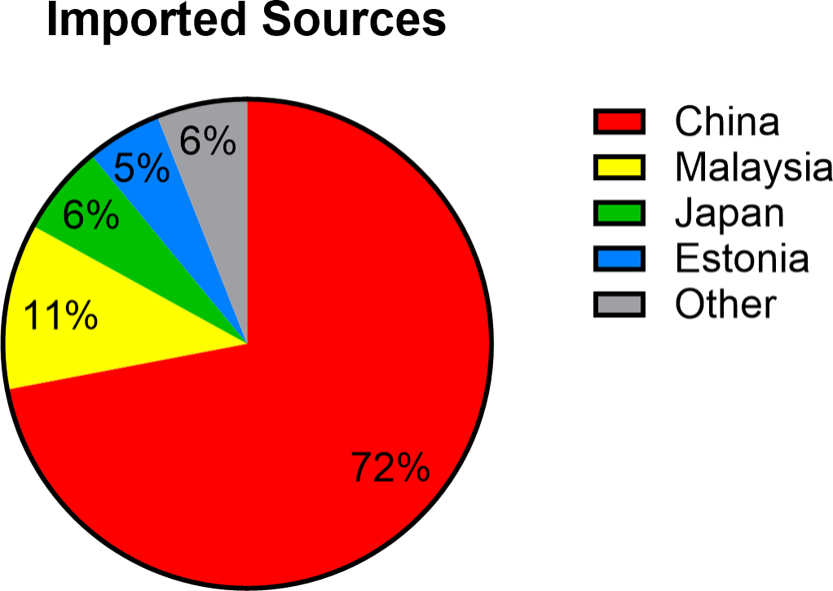
Percentage of imported REE resources into the U.S. from 2019 to 2022.^9^

In North America, there is 17.6 million tons of indicated REE sources available, 3.6 million tons in the U.S. and 14 million tons in Canada,^9^ so although there is not a short supply of REEs in the Earth’s crust, they exist at relatively low concentrations. Figure 2 provides the average composition of rare earth oxides (REOs) at various deposits.^15^ Heavy rare earth elements (Gd–Lu) plus europium exist at particularly low concentrations, less than 1 wt %.^15^

**Figure 2:**
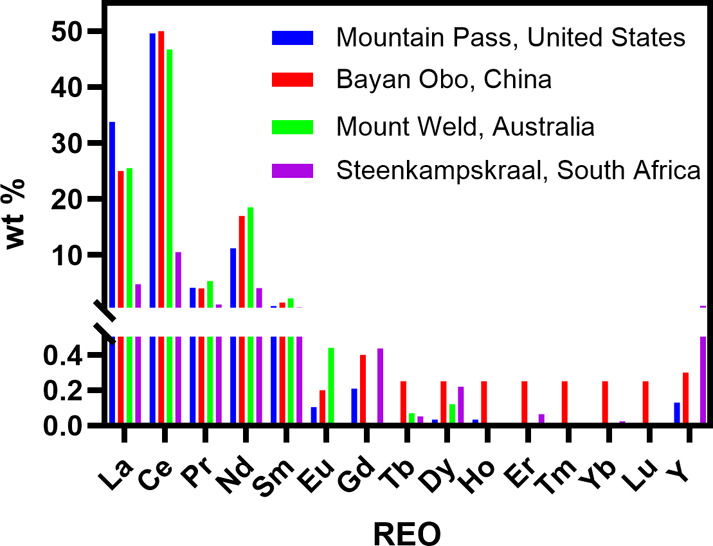
Average rare earth oxide (REO) compositions (wt %) in Mountain Pass concentrate (California, USA), Bayan Obo ore (Mongolia, China), Mount Weld ore (Southwestern Australia), and the Steenkampskraal deposit (South Africa).^15^

They have been deemed “rare” because they are difficult to separate from one another due to similar intrinsic properties like ionic radii and oxidation number.^1,16^ Small differences in these properties are what most separation techniques use for recovery. The common techniques that have been developed to study the separation of REEs include ion exchange chromatography, electrolysis, distillation, precipitation, and solvent extraction.^16–21^ In larger-scale/industrial separation processes, liquid–liquid extraction, also known as solvent extraction, is currently the most common method for obtaining individual REEs.^22^ Liquid–liquid extraction, uses two liquid phases, a more polar (MP) phase which is typically aqueous and a less polar (LP) phase that is usually an organic solvent.^23^ Generally, the aqueous phase contains the metal ion(s), and upon mixing, metals ions transfer across the phase boundary into the organic phase where selective ions bind to the extractants and reside as a metal–ligand complex. This type of system has been termed traditional liquid–liquid extraction (T-LLE). Modifications to solvent type, ligand design, pH and the concentration of acid, metal, and ligand are often varied to improve extraction efficiency or selectivity. Common ligands used in liquid–liquid extraction systems for the separation of REEs are reported in Figure 3.^11,24^

**Figure 3:**
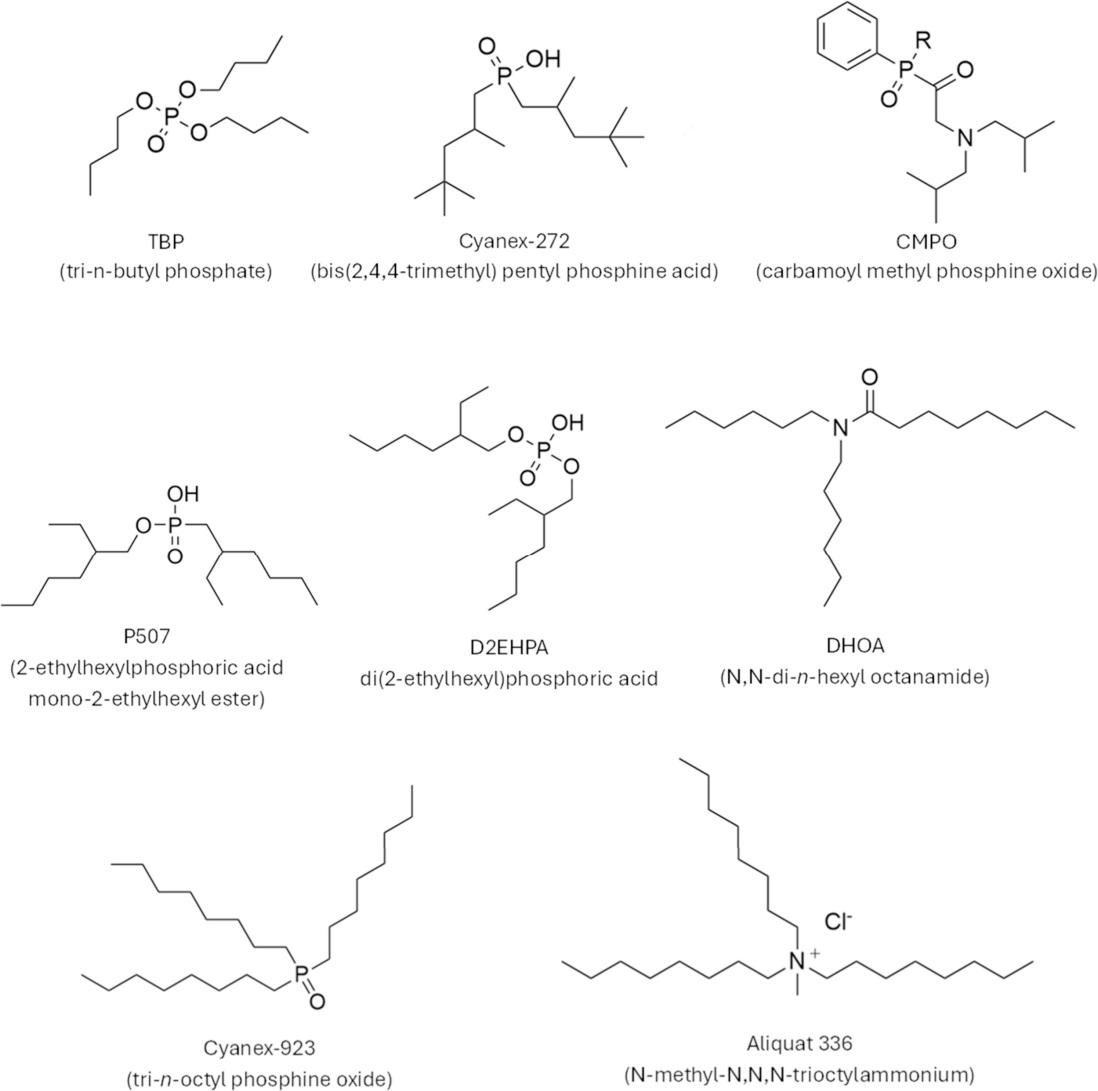
Common ligands used in T-LLE systems for REE separation.^11,24^

Minor structural changes to extractants have a significant influence on selectivity and extraction/separation efficiency of metals, and the solubility of extractants is a large deciding factor when choosing the appropriate extractant for a system. Most extractants that have shown selectivity for REEs are nonpolar and soluble in organic solvents such as *n*-heptane, kerosene, dichloromethane, hexanes, and toluene. These solvents have high lipophilicity and volatility causing them to be harmful to the environment and human health^25,26^ and when used in industrial-scale solvent extractions, large amounts of media are required. At a facility processing 18000 tons of aqueous solution containing rare earth oxides annually, resource consumption includes approximately 273 tons of kerosene, 120 tons of the extraction agent di­(2-ethylhexyl)­phosphoric acid (D2EHPA), 227000 m^3^ of water, 34000 tons of hydrochloric acid and operation requires over 6.9 million kWh of electricity.^27^ Facilities storing these large volumes of kerosene and organic extractants are conducive to severe fire hazards putting the facility, employees, and environment at risk. Additionally, the substantial generation of contaminated and hazardous waste at these facilities requires extensive waste treatment, leading to higher operational costs and increased environmental concerns for the surrounding areas.

For these reasons, modifications to the T-LLE methods are being explored. Literature on solvent extraction systems without organic media or without aqueous media and solvent extractions where multiple diluents or extractants are added is being reported. Data on the number of peer-reviewed article publications was collected using Web of Science (Figure 4) and shows the overall increasing trend in these areas since 2010 suggesting recent and future interest in aqueous two-phase systems, nonaqueous systems, and synergistic solvent extraction. There has not been a review, to our knowledge, which summarizes these modified T-LLE systems (e.g., ATPS, NAS, and synergistic systems) and compares them to each other or to T-LLE, the industry standard. This review focuses on these three solvent extraction systems, since it is necessary to summarize these advances in the area of REE separation. Additionally, it is important to evaluate the TRLs of these systems, as the goal is to implement them into industrial settings. We also report ongoing and future work in another area of selective REE separation that is based on a less considered intrinsic property of REEs, magnetic susceptibility.

**Figure 4:**
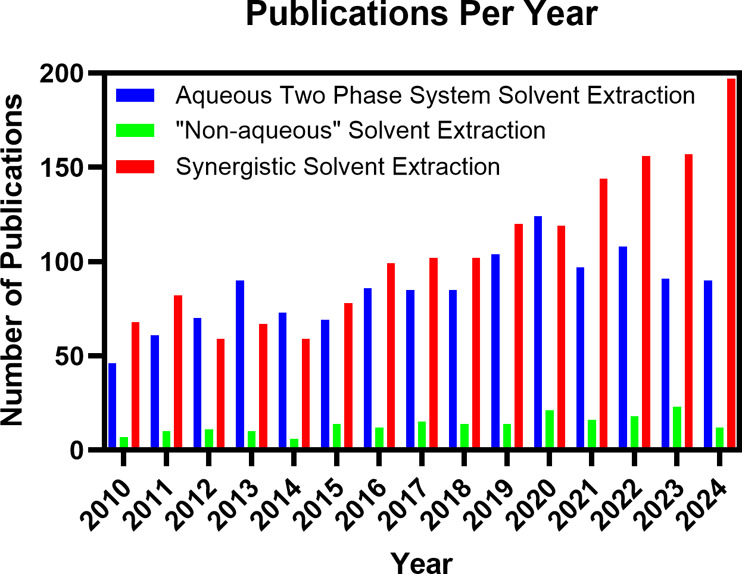
Report of the number of publications per year for an aqueous two-phase system solvent extraction, nonaqueous solvent extraction, and synergistic solvent extraction.

### 1.1 Theory

During any metal extraction process containing one or more ligand types and two immiscible phases, transport across a phase interface occurs by first coordinating a metal salt (*MX*_*X*_), metal cation (*M* ^*n+*^), or metalate anion (*MX*_*X*_^*n–*^) with ligands.^28^ Ligands will bind to the metal either in the outer sphere as an ion pair or in the inner coordination sphere where they form more stable neutral complexes by being directly attached to the metal. Ultimately, it is the coordination chemistry of the metal and ligand that determines the extractability and mechanism of the complex from the MP phase to the LP phase (denoted as the aqueous and organic phases in eqs 1–3). When metal salts (*MX*_*X*_) are present in the aqueous phase, they generally form neutral complexes in the organic phase (eq 1). The ligand may bind in the inner or outer sphere, but ligands with highly selective extraction will reside in the inner sphere.^28^ Equation 2 is the general mechanism for metal cations in the aqueous phase and an anionic ligand which usually binds in the inner coordination sphere.^28^

1 $$\begin{array}{c} nL_{\left(\mathrm{org}\right)}+MX_{X}⇌{\left[MX_{X}{\left(L\right)}_{n}\right]}_{\left(\mathrm{org}\right)} \end{array}$$

2 $$\begin{array}{c} nLH_{\left(\mathrm{org}\right)}+M^{n+}⇌{\left[M{\left(L\right)}_{n}\right]}_{\left(\mathrm{org}\right)}+nH^{+} \end{array}$$

Metalate anions can exist in the aqueous phase when there are high concentrations of the counteranion in the surrounding environment. When this occurs, a metalate anion usually forms a charge-neutral complex in the organic phase with a cationic extractant (eq 3) in the outer coordination sphere.^28^

3 $$\begin{array}{c} nL_{\left(\mathrm{org}\right)}+nH^{+}+MX_{X}^{n-}⇌{\left[{\left(LH\right)}_{n}MX_{X}\right]}_{\left(\mathrm{org}\right)} \end{array}$$

When two immiscible solvents are in contact, both contain a solute of the same molecular condition (chemical form), and have a constant temperature, we can consider Nernst’s distribution law, which states that the ratio of the solute in the two solvents is a constant known as the partition coefficient (*P*), distribution coefficient (*K*_d_), or distribution ratio (*D*_*A*_). The distribution of the solute (metal) in the two phases is generally what is used in literature for reporting extraction results, and it is determined using eq 4:

4 $$\begin{array}{c} D_{A}=\frac{{\left[A\right]}_{LP}}{{\left[A\right]}_{MP}} \end{array}$$

where [*A*]_*LP*_ and [*A*]_*MP*_ are the concentrations of solute A in the respective phases.^29^

However, it is rare that the metals are in one particular molecular condition in both phases, so we must consider the distribution ratio, *D*_*M*_. This is a ratio of the sum of concentrations of a metal, regardless of the chemical form, in the LP phase to the sum concentration in the MP phase as seen in eq 5:

5 $$\begin{array}{c} D_{M}=\frac{\underset{j}{\sum }{\left[M_{j}\right]}_{LP}}{\underset{j}{\sum }{\left[M_{j}\right]}_{MP}} \end{array}$$

where [*M*_*j*_]_*LP*_ and [*M*_*j*_]_*MP*_ are the concentrations of the *j*th chemical form of the metal in the less polar phase and the more polar phase.^30^

Extraction results from aqueous metal feeds are sometimes reported as a % extraction (%*E*) using eq 6.

6 $$\begin{array}{c} \%E=\left(\frac{{\left[M\right]}_{{\mathrm{aq}}_{0}}-{\left[M\right]}_{{\mathrm{aq}}_{f}}}{{\left[M\right]}_{{\mathrm{aq}}_{0}}}\right)\times 100 \end{array}$$

­[*M*]_aq_0__ and [*M*]_aq_f__ are the initial and final concentration of the metal in the aqueous phase, or a separation factor (SF_*A*/*B*_) when looking at two metal solutes (eq 7),

7 $${\mathrm{SF}}_{A/B}=\frac{D_{M_{A}}}{D_{M_{B}}}$$

where *D*_*M*_ is the distribution ratios of metals A and B.^29,30^

Lastly, when we start to discuss synergistic effects, we should consider the synergistic enhancement coefficient, described as *R* (eq 8) or SC (eq 9), which can be expressed as

8 $$\begin{array}{c} R=\frac{D_{\mathrm{mix}}}{D_{1}+D_{2}} \end{array}$$

9 $$\begin{array}{c} \mathrm{SC}=\log\frac{D_{\mathrm{mix}}}{\left(D_{1}+D_{2}\right)} \end{array}$$

where *D*_mix_ represents the distribution ratio of the mixed reagents (1 + 2), and *D*_1_ and *D*_2_ represent the distribution ratios while extracting with the extractants separately (1 or 2).^31–33^ Both *R* and SC can be used to quantify the effect of a synergist on the extraction of a target metal and determine if the combination of an extractant and a synergist enhances extraction beyond the primary extractant alone.

More recently, Gómez-Sánchez et al. reported a new marker for the enhancement or diminishment of synergistic solvent extraction called the relative distribution enhancement factor, *H* (eq 10). They show how using *R* to report synergistic enhance can be misleading when varying extractant ratios compared to individual extractants, and it is limited to two extractants.^34^ *H* is expressed as

10 $$\begin{array}{c} H=\frac{D_{\mathrm{mix}}}{D_{H}} \end{array}$$

where *D*_*H*_ is the distribution ratio of the reagent with the highest extraction efficiency.^34^ When *H* > 1 the mixture of reagents enhances extraction, when *H* = 1 there is no enhancement, and when *H* < 1 the mixture of reagents diminishes extraction.^34^ This expression can also be used for systems with more than two extractants.

## 2 Aqueous Two-Phase Systems

Aqueous two-phase systems (ATPS), sometimes called aqueous biphasic systems (ABS), have been studied since the 1950s primarily for the extraction of biomolecules such as proteins, peptides, and nucleic acids.^35^ In metallurgy, ATPS is being developed because of environmental concerns involving large volumes of organic solvents in T-LLE. ATPS are generally ternary systems that form two phase like regions rich in the respective reagents at a certain set of conditions (composition, temperature, and pressure). These ternary systems consist of water and two water-soluble reagents. The water-soluble reagents can be a combination of polymers, salts, sugars, surfactants, carbohydrates, and ionic-liquids,^35^ but the use of ATPS for extraction of REEs is a relatively new objective, so most combinations of water-soluble reagents have not been studied. The major focus of ATPS for REE acquisition has been the utilization of an ionic liquid (IL) in one of the phases and therefore is what is mentioned in this section.

### 2.1 Ionic Liquid Based ATPS

The development of ILs as a replacement for the organic phase has been a major focus for those working on green approaches to solvent extraction. ILs are nonmolecular solvents composed of cation/anion pairs with melting points below 100 °C.^36^ They are considered organic salts, meaning they are composed of organic cations (e.g., imidazolium or methyl acetate-based cations) and either organic anions (e.g., bis­(trifluoromethylsulfonyl)­amine ([Tf_2_N]^−^)) or inorganic anions (e.g., chloride ([Cl]^−^), hexafluorophosphate ([PF_6_]^−^)). They are green replacements due to their nonflammability and low or negligible vapor pressure. Additionally, they have high thermal stabilities, and tunable hydrophobicity with adjustment of the cation/anion pair.^11,37^ There are two main types of ILs: aprotic and protic. Aprotic ILs are fully ionic and are not generated by the donation of a proton. Protic ILs are also fully ionic but are produced from an acid and base and involve a proton transfer (eq 11).^38^

11 $$HA+B\to A^{-}+HB^{+}$$

ILs can be used as a solvent alternative while also exhibiting extractant behavior. There must be two immiscible phases to carry out solvent extraction, so the ILs must be hydrophobic in nature or hydrophilic with the addition of a salting-out agent that aids in creating two phase like regions. In this section, ILs are being used to develop green alternatives without organic solvents (ILs being used as replacements for the aqueous phase and as purely extractants or synergist are discussed in subsequent sections).

The simplest IL-based solvent extraction system uses an IL as the extractant and diluent, so no organic ligands or additional functionalized (or nonfunctionalized) ILs are needed for metal transfer across phase interfaces. Chen et al. reports a ternary ATPS with tetrabutylphosphonate nitrate ([P_4444_]­[NO_3_]) as a hydrophilic IL, NaNO_3_ as the salting out agent and water.^39^ Nd­(III) was selectively extracted from the salt-rich phase into [P_444_]­[NO_3_] with a %*E* > 99% and a SF of 10^3^ from cobalt­(II) and nickel­(II).^39^ The amount of IL in the system had no effect on %*E*, but the amount of salt had a significant influence.^39^ It is an advantage that this system is dependent on the amount of NaNO_3_ since the IL must be synthesized, and salts can be obtained at a relatively low cost. A similar simple system was used in a series of extraction steps for recovery of Ce­(IV) from La­(III), Nd­(III), and Pr­(III) except the ILs were hydrophobic and paired with an aqueous nitric acid (HNO_3_) phase.^21^ The concentration of HNO_3_ had a significant impact on the extraction selectivity, suggesting these systems can be easily modified by the acid or salt concentration for enhanced extraction efficiency.

Li et al. used a simple IL-based ATPS to focus on binary metal mixtures containing one REE and one transition metal. The transition metals were extracted into the IL phase, while the respective REE was left behind in the salt-rich phase (Nal, LiCl, or HCl) separating REE/transition metal pairs: Sm/Co, Nd/Fe, Eu/Zn, and La/Ni. After obtaining good separation of Sm (III) from Co (III), the effect of the salt was evaluated.^40^ By varying the concentration of all three salts, it was found that different metal pair separations were maximized with specific salts and salt concentrations.^40^ Figure 5 displays the effect of the concentration of NaCl and LiCl on the distribution ratios of some lanthanides and transition metals. The transition metals were efficiently extracted into the IL at various salt concentrations whereas the lanthanides had low distribution ratios (*D* < 10) for all considered salt concentrations.

**Figure 5:**
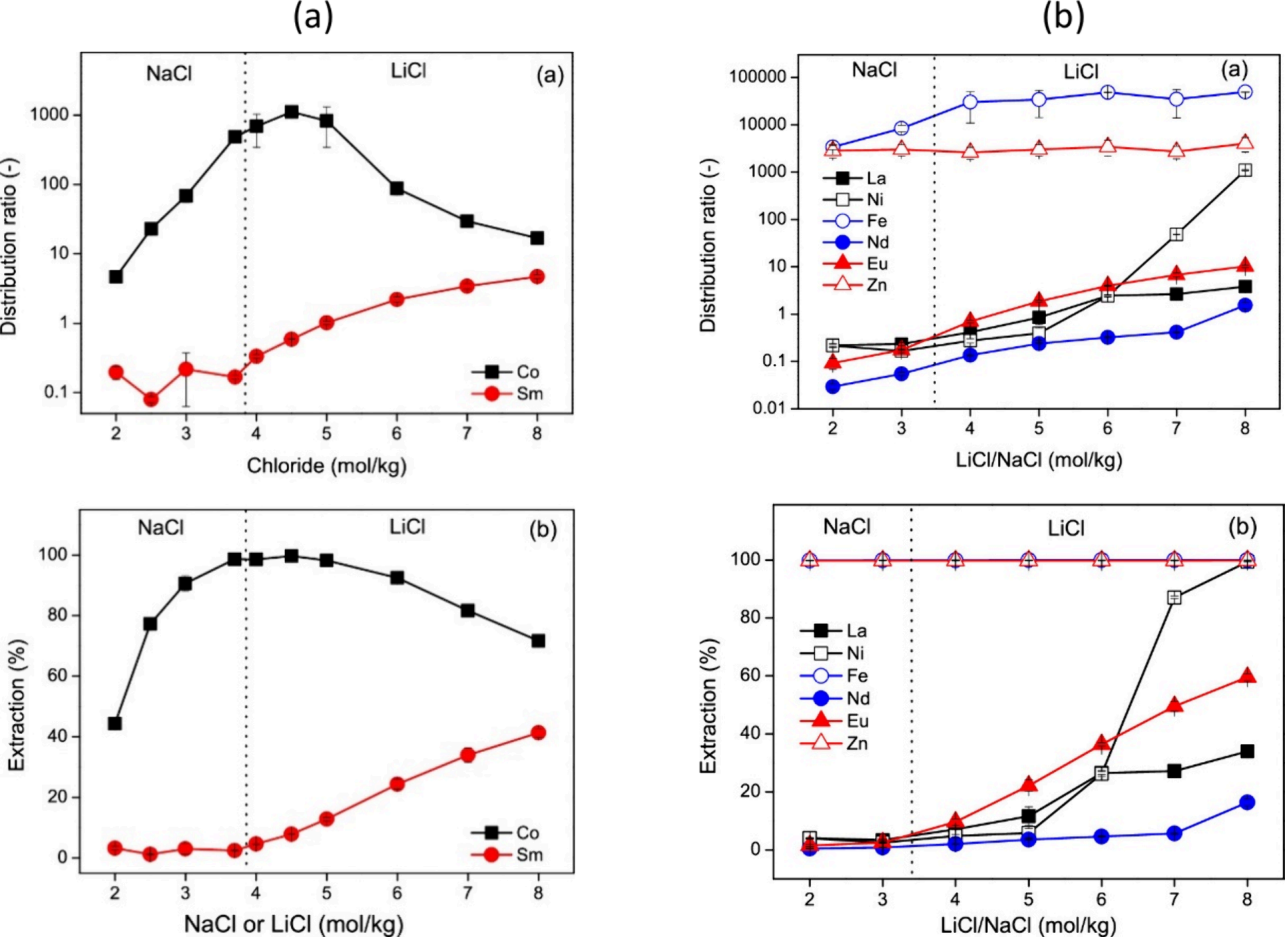
Distribution ratios and percent extraction as a function of salt concentration on (a) cobalt and samarium (b) lanthanum, nickel, iron, neodymium, europium, and zinc.^40^ Reproduced from ref 40. Copyright 2022 American Chemical Society.

This phenomenon, known as the salting-out effect, greatly impacts solvent extraction systems, leading to applications in hydrometallurgy.^41–43^ The hydration capability of a salt anion and its metal complexation ability mainly determine the system’s salting out behavior. It was found that the solubility of ILs follow the Hofmeister series, a solubility trend first applied to proteins.^41–44^ Adding a salting out agent decreases the solubility of the IL, generating two phases that are better for transport across the liquid–liquid interface. Although these effects depend on multiple parameters (the nature of the two phases and their concentrations) in each system, the anion of the IL has the greatest impact on how a salting-out agent might affect solubility.^41^ Poor hydration of the anion enhances extraction by forming stable complexes with the metal and providing better transport from the MP phase to the LP (IL) phase.^41,44^ Salts of higher cationic charge also exhibit stronger salting out abilities.^42^ Several reports are in agreement that low salt concentrations result in a salting-in region and high salt concentrations result in the IL salting-out.^41,43^ The salting-in effect is desirable when ILs are involved in the extraction mechanism and enhanced metal transfer is needed, and the salting-out effect is desirable for increased phase separation.

Sometimes, organic extractants are added to ILs just like in T-LLE. For example, Cyanex 923, a ligand known to be selective for REEs, was employed in a systematic evaluation for the extraction of Nd­(III) from HNO_3_ into ILs of different hydrophilicities. The hydrophobicity of an IL can be altered by changing the length of the alkyl chain of the cation of the IL which changes the hydrophobicity of the IL as a whole. Here, bis­(trifluoromethylsulfonyl)­imide ([Tf_2_N]^−^) was kept as the anion and the cation was varied.^45^ They found that [Tf_2_N]^−^ paired with the small hydrophilic cations, [C_4_mim]^+^ and [N_1444_]^+^, were much more efficient than the hydrophobic cations, [C_10_mim]^+^ and [N_1888_]^+^.^45^ They determined that the IL was involved in the extraction mechanism, so the low solubility of the hydrophobic ILs in the aqueous phase inhibited metal transfer. However, increasing the nitrate concentration in the aqueous phase increased the extraction efficiency of the hydrophobic cations due to nitrate ion involvement in the extraction mechanism as seen in eqs 12 and 13.^45^

12 $$\begin{array}{c} {\mathrm{Nd}\left({\mathrm{NO}}_{3}\right)}^{2+}+n\mathrm{L̅}+2\overset{®}{Q\left[{\mathrm{Tf}}_{2}N\right]}⇋\overset{®}{\mathrm{Nd}L_{n}{\left({\mathrm{NO}}_{3}\right)\left({\mathrm{Tf}}_{2}N\right)}_{2}}+2Q^{+} \end{array}$$

13 $$\begin{array}{c} {\mathrm{Nd}({\mathrm{NO}}_{3}{)}_{2}}^{+}+n\mathrm{L̅}+\overset{®}{Q\left[{\mathrm{Tf}}_{2}N\right]}⇋\overset{®}{\mathrm{Nd}L_{n}({\mathrm{NO}}_{3}{)}_{2}\left({\mathrm{Tf}}_{2}N\right)}+Q^{+} \end{array}$$

This implies the sensitivity of these systems to the hydrophobicity of the IL depending on the extraction mechanism. Hydrophilic cations increase mobility for contact with the metal in the aqueous phase but phase immiscibility becomes a concern.

Often, the hydrophobicity of an IL is tuned by adjusting the length of any alkyl chains on the cation. There is evidence of both shorter and longer alkyl chains improving extraction of selective REEs.^46–49^ Small increases in the alkyl chain length increase hydrophobicity of the IL rich phase and help stabilize the metal complex in the IL phase, preventing transfer back into the MP (aqueous) phase. This was the case for the extraction of Ce­(III) by a simple imidazolium based IL, 1-butyl-3-methylimidazolium chloride (C_4_MIMCl).^49^ After comparing C_4_MIMCl with C_2_MIMCl (Figure 6), it was determined that, indeed, the IL with the longer alkyl chain (C_4_MIMCl) increased the extraction efficiency of Ce­(III) due to an increase in the hydrophobicity of IL phase.^49^ In Figure 6, the percent extraction of Ce­(III) increases from approximately 5% extraction with C_2_MIMCl (right) to 34% extraction with C_4_MIMCl (left). The same goes for a system extracting Sc­(III) with two types of quaternary ammonium based IL, [N333 MeOAc]­[Tf_2_N] and [N444 MeOAc]­[Tf_2_N], where the alkyl chains are propyl and butyl groups.^47^ The IL with the butyl alkyl chain ([N444 MeOAc]­[Tf_2_N]) exhibited stronger hydrophobicity and, therefore, higher extraction of Sc­(III) under all explored conditions (pH and IL concentration).^47^ The opposite effect occurred in a system comparing ILs with three different alkyl chain lengths: ([C_*n*_MIM]­[Tf_2_N], *n* = 4, 8, and 12). In this case, the IL with the shortest alkyl chain, [C_4_MIM]­[Tf_2_N], had the highest extraction of Y­(III) and Eu­(III). However, the extraction ability of this IL compared to the longer alkyl chains could not be explained and was left to peculiar solvent effects.^46^

**Figure 6:**
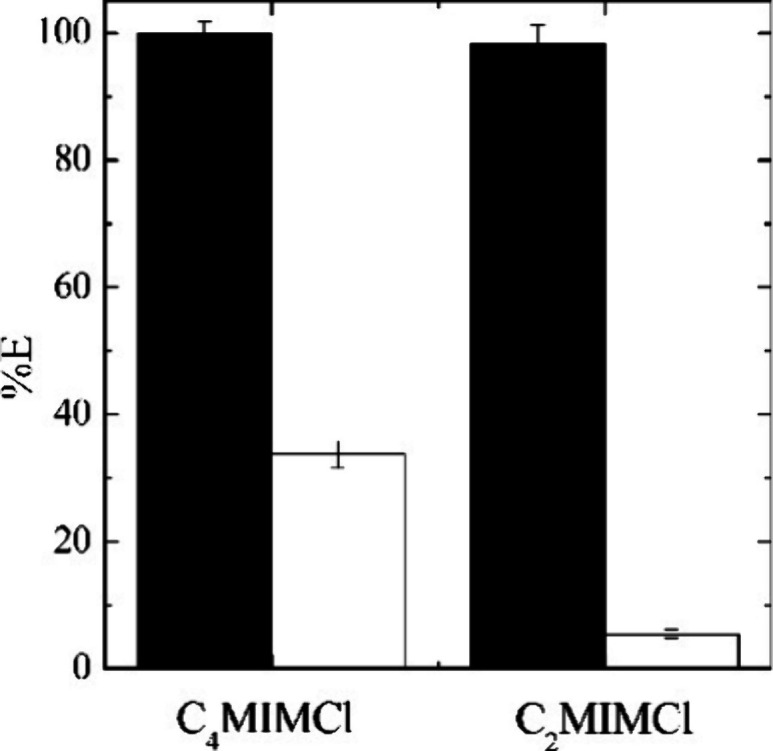
Effect of two different ILs (C_4_MIMCl and C_2_MIMCl) on the percent extraction of La­(III) (solid black bar) and Ce­(III) (empty bar) in an ATPS containing the IL, K_2_CO_3_, and H_2_O.^49^ Reproduced with permission from^49^. Copyright 2019 American Chemical Society.

Adjusting the anion type can also influence the hydrophobicity of the IL. It is ideal for ILs in ATPS to be nonfluorinated as they are less expensive and safer than popular fluorinated ILs such as hexafluorophosphate ([PF_6_]^−^) and bis­(trifluoromethylsulfonyl)­amine ([Tf_2_N]^−^) that give efficient extraction due to strong hydrophobicity without too high of viscosities.^39^ Nonetheless, fluorinated ILs have potential hazards associated with them due to possible conversion to hydrofluoric acid.^50^ Since developing green extraction methods is generally the motivation for employing ILs versus organic solvents, nonfluorinated alternatives are the better option. However, the high viscosity of nonfluorinated ILs in their undiluted, pure form promotes slow kinetics and longer mixing times which becomes a challenge for developing extraction systems.^50–52^ For instance, a viscous (>20000 cP) nonfluorinated carboxyl-functionalized phosphonium IL was paired with NaCl and water for the extraction of Sc­(III).^50^ In order to minimize the effects of high viscosity, the IL was presaturated with 16 wt % NaCl before mixing it with the aqueous feed solution. Performing extraction experiments at higher temperatures is another method for decreasing viscosity of the IL. When the temperature of an IL, trihexyl­(tetradecyl)­phosphonium nitrate (Cyphos IL 101), loaded with samarium and cobalt was increased to 60 °C from room temperature, the viscosity decreased from 6630 cP to 350 cP resulting in an equilibrium time of approximately 20 min.^53^ It is unclear how the overall percent extraction and selectivity changed as the temperature increased, but the percent extraction of samarium was nearly 100% *E* after 20 min at 60 °C.^53^ Another nonfluorinated IL, tributylmethylammonium nitrate ([N_4441_]­[NO_3_]), was implemented in an ATPS to target REEs from mixtures of other metal contaminants, specifically transition metals (Fe­(III), Ni­(III), and Co­(II)).^52^ The design of this IL is what resulted in ultralow viscosity and a high extraction percentage of Nd­(III) after just 10 s.

A new class of ionic liquids known as pseudoprotic ionic liquids (PPILs) are also being used in ATPS as extractants and replacements for volatile organic phases.^54–58^ PPILs are made up of an acid and base, like protic ILs, but they are not fully ionic because their reaction involves partial ionic and hydrogen bonding.^54^ For instance, a PPIL was generated from the reaction of Primene 81-R, a primary amine, and Cyanex 572, an acidic compound already common in extraction of REEs.^58^ Undiluted PPILs have been combined with equal volumes of an aqueous phase to give excellent, quantitative recovery of REEs.^57^ This suggests that PPILs have the potential to be generated with extractants already integrated into industrial separation processes as an alternative to current systems.

Parallel comparisons of aqueous/IL and aqueous/organic systems have been reported to indicate the proficiency of IL-based ATPS compared to T-LLE. When imidazolium-based ILs were used an as alternative to *n*-dodecane in an identical system, extraction performance increased for quantitative recovery of Y­(III) and Eu­(III).^46^ More recently, Turanov et al. did a parallel comparison by substituting 1,2-dicholoethane with methyltrioctylammonium bis­(trifluoromethanesulfonul)­imide ([N_1888_]­[Tf_2_N]) for enhanced extraction efficiency across the entire lanthanide series (excluding Pm­(III)) plus Y­(III).^59^

Overall, the amount of ATPS for REE recovery is limited, and other combinations of water-soluble reagents besides ILs should be further explored. For example, polymer/salt and surfactant/salt systems have been implemented for transition metal recovery,^60,61^ and surfactant/IL for metalloids.^62^ ATPS also has shown potential to be used for decontamination of used materials during REE production.^51^

## 3 Nonaqueous Systems

Expanding the use of ATPS is great for limiting flammable, volatile solvents but can increase the generation of contaminated wastewater that must be treated. Treatment of contaminated water is 20–30% of REE production cost and would only be expected to increase with ATPS.^11^ Solvent extractions by nonaqueous systems (NAS) can partially resolve the issue of water over consumption and generation of wastewater associated with ATPS. In this area, a system is considered nonaqueous when the volume % water is less than 50%.^63^ Nonaqueous extraction methods can increase selectivity and minimize extraction steps leading to lower energy consumption.^63^ Most NAS fall under the recently developed field of solvometallurgy, a branch of extractive metallurgy, where both phases are organic or inorganic. Not all NAS should be considered solvometallurgy because the solvents must be nontoxic (not flammable or volatile like many organic solvents used in T-LLE such as dichloromethane or chloroform). Binnemans et al. provides a list of undesirable, usable, and preferred molecular organic solvents in solvometallurgy (Table 2).^63^ Deep eutectic solvents (DES), ILs, and inorganic solvents are also applicable to solvometallurgy, but inorganic solvents are generally too reactive and toxic to be utilized.^63^

**Table 2:** Organic Solvent Guide for Solvometallurgy Solvent Selection^a^

preferred	usable	undesirable
water	cyclohexane	pentane
methanol	methyl cyclohexane	hexane(s)
ethanol	heptane	diisopropyl ether
2-propanol	isooctane	diethyl ether
1-propanol	toluene	dichloromethane
1-butanol	xylenes	dichlorocthane
*tert*-butanol	acctonitrile	chloroform
1-octanol	2-methyltetrahydrofuran	dimethylformamide (DMF)
glycerol	cyclopentyl methyl ether	pyridine
polyethylene glycol (PEG)	dimethyl sulfoxide (DMSO)	*N*-methylpyrrolidone (NMP)
polypropylene glycol (PPG)	ethylene glycol	dimethoxyethane (DME)
acetone	acetic acid	dioxane
methyl ethyl ketone (MEK)	methanesulfonic acid	tetrahydrofuran (THF)
methyl isobutyl ketone (MIBK)		petroleum spirits
diisobutyl ketone (DIBK)		benzene
γ-valerolactone		nitrobenzene
ethyl acetate		carbon tetrachloride
isopropyl acetate		carbon disulfide
isobutyl acetate		
isoamyl acetate		
dimethyl carbonate		
diethyl carbonate		
propylene carbonate (PC)		
propylene glycol ethers		
propylene glycol others acetates		
biodiesel		
(biodegradable) GTL solvents^a^		

a Reproduced from ref 63. Available under a CC-BY 4.0 license (http://creativecommons.org/licenses/by/4.0/). Copyright 2017 Koen Binnemans and Peter Tom Jones.

b GTL = gas-to-liquid.

For the extraction of REEs, NAS have been assembled by four main solvent combinations: organic/organic, IL/organic, IL/IL, and DES/organic. There are few NAS that have been designed for REE selectivity, and most have considered the effect of using ethylene glycol (EG) as the MP phase in organic/organic set-ups.^64–69^ Batchu et al. utilized EG containing lithium nitrate (LiNO_3_) and Cyanex 923, another ligand with known selectivity of REEs, and *n*-dodecane to separate heavy REEs and light REEs.^64^ A similar system, but with LiCl instead of LiNO_3_, showed selectivity for Sc­(III), Yb­(III), and Y­(III), and they evaluated the effect of adding other organic solvents (50% (v/v)) to the MP phase and found that certain solvents, including dimethyl sulfoxide, added to EG enhanced separation factors.^65^ This system has also been used to extract Y­(III) and Eu­(III) from each other at concentrations comparable to fluorescent lamp waste compositions, and it was applied to mixer-settler experiments to confirm the potential of these systems on a larger scale.^66^ Other selective metal extractions from EG solutions have shown selectivity for Gd­(III) and Nd­(III) with Cyanex 572,^67^ and Co­(II) from Sm­(III) with Aliquat 336.^69^ Polyethylene glycol 200 (PEG 200) has also been used for organic/organic setups for the extraction of Dy­(III) and Nd­(III) simultaneously. However, the aim was to separate Dy­(III) from Nd­(III) with the intention to recycle NdFeB magnets, so in order to separate the two ions, they found that when small amounts of water were added to the PEG 200 phase, Dy­(III) was able to be separated from Nd­(III) with Cyanex 923 and another commercial extractant, P350.^70,71^ This system is still considered a nonaqueous solvent extraction since the volume % water is less than 50%.^63^

There are few reports of ILs being used in the NAS for REE separation. One being a recent comparison of an organic/organic and IL/organic system with an extractant in the respective phases showed that the chloroform/EG system was more efficient at extraction Gd­(III) than the IL/EG system (IL= [C_1_C_10_im^+^]­[Tf_2_N^–^]). However, the IL/EG system could still recover all 4f-ions when the extractant was introduced into the IL phase.^72^ As previously mentioned, extractants are not always necessary when ILs are involved in the systems, as they can act as both the extractant and diluent. After a system with two immiscible ILs (IL/IL) were used for separation of cobalt and nickel, the same system was used for separation of REEs from nickel.^73,74^ The MP phase was an undiluted imidazolium-based IL containing the metal ions that were in contact with undiluted trihexyl­(tetradecyl)­phosphonium bis­(2,4,4trimethylpentyl)­phosphinate (Cyphos IL 104). Selectivity toward REEs across the series with the highest distribution ratio for Yb­(III) was the result of this IL/IL system.^74^ Additionally, they showed rare earth oxides exhibited high solubility in a solution containing a small amount of concentrated HCl in an imidazolium-based IL suggesting the use of this system for leaching REEs from primary or secondary sources in addition to solvent extraction.^74^

DES can also be combined with a desirable organic solution for the NAS. DES are commonly compared to ILs because of analogous behavior and properties.^75^ Specifically, DES are liquids which have an elemental composition that results in a low melting point (eutectic composition) which is around room temperature (Figure 7).^75^ DES do not have to contain ionic components, unlike ILs, as pairing nonionic hydrogen bond acceptors and hydrogen bond donors can be considered DES, but DES are most commonly generated from a quaternary ammonium salt and a hydrogen bond donor or metal chloride.^76^ There are advantages to choosing DES over ILs as DES are less expensive and easier to synthesize.^76,77^ However, the number of extraction systems with DES is currently limited, and there are only a few that have recovered REEs in NAS. In 2017, Riaño et al. separated a Dy/Nd mixture by extracting Dy (III) into an organic phase which contained toluene and an extractant (either Cyanex 923 or D2EHPA) from a choline chloride and lactic acid DES.^77^ This system shows high potential for large scale REE processing as they applied this system to a small mixer-settler and saw no issues with the viscosity of the DES at ambient temperatures, unlike many undiluted hydrophobic ILs.^77^ A levulinic acid-choline chloride (LevA-ChCl) DES and pure levulinic acid (LA) were compared for the leaching and solvent extraction (paired with aromatic molecular solvents) of REEs from lamp phosphor waste.^78^ Both solvents gave comparable, efficient results for the leaching and solvent extraction (with D2EHPA) of the yttrium oxide phosphor (Y_2_O_3_:Eu^3+^), but it was concluded that LA was the ideal solvent since the DES exhibited calcium precipitation from the lamp phosphor waste and large amounts of pure LA can be generated from byproducts from sugar production making it a more green solvent than the DES.^78^ DES/IL and DES/aqueous systems^79,80^ have been used for transition metal separation but there is no evidence of these systems for REEs. Additional DES investigations should be conducted, as they have similar beneficial properties as ILs but also exhibit advantages (i.e., cost and availability) that make them more applicable to industrial scale processes.

**Figure 7:**
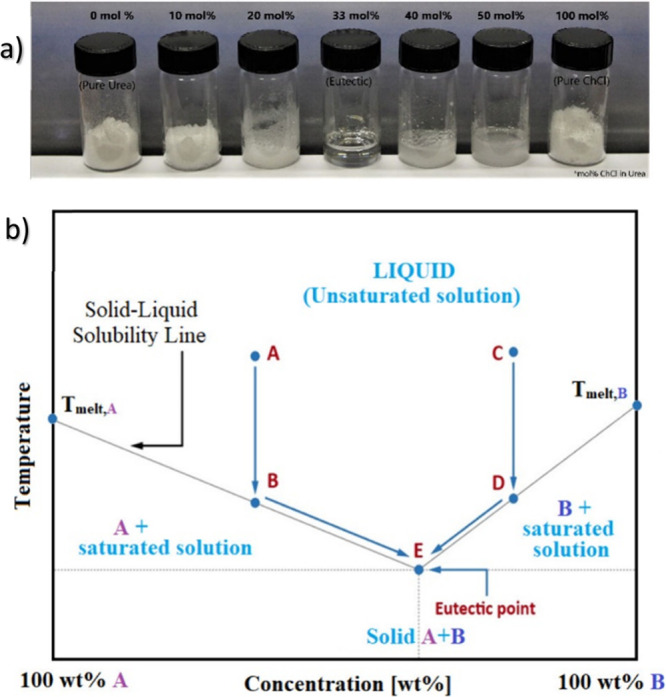
(a) Mixtures of choline chloride and urea at 303 K, demonstrating the eutectic composition of the mixture (33 mol % choline chloride). (b) Schematic phase diagram for eutectic binary mixtures.^75^ Reproduced with permission from^75^. Copyright 2014 American Chemical Society.

## 4 Synergistic Effects

Ligand design and concentration, diluent types, volume ratios, pH, and number of extractants/synergists have been explored for metal extraction enhancement. Ligand design and solvent choice have thoroughly been investigated for REE processing, but there has been recent increased interest in understanding the synergistic effects of combining other physiochemical methods to enhance separation. Here we summarize recent REE synergistically enhanced extractions by discussing the effects of combining extractants in the LP phase and auxiliary agents in the MP phase.

When an additional extractant is added to the organic (LP) phase of a T-LLE at an appropriate concentration, extraction efficiency can be enhanced. Due to the coordination number of 4f ions in solution (8 or 9), water molecules often bind to REE ions and decrease their solubility in the LP phase. The addition of synergistic agents can counter this effect and increase the solubility of the REE complex in the nonaqueous phase (organic or IL diluent) by taking the place of any bound water molecules.^33,81^ Most combinations of primary and synergistic extractants are acidic/neutral. The primary extractant will have stronger coordination that the synergistic agent.^33^ Other combinations exist but have not yet, to our knowledge, been implemented into REE separation processes: acidic/acidic, cationic/neutral, cationic/anionic, and two cationic elements.^82^ Depending on the mixture of elements, concentrations of extractants, and the extraction mechanism, both synergistic and anti-synergistic (also called antagonistic) effects can occur.^82^ Antisynergism happens when the addition of a second extractant decreases extraction efficiency or separation compared to when the metallic species is extracted with the primary extractant alone. It has been explained that when the synergistic agent is added in excess it increases its interaction with the primary extractant, decreases the interaction with the metal ion, and results in diminished transfer of the metal into the LP phase, but multifaceted systems can be more complex.^82^ Referring to eq 9, synergism dominates when SC > 0, and when SC < 0, the addition of the synergist agent causes an anti-synergistic effect.^33^

The addition of synergist to T-LLE systems for select REEs across the lanthanide series has shown favorable results. Dashti et al. compared extraction efficiencies of systems containing single extractants (Cyanex 572 or Ionquest 801) with a mixture of the two.^83^ Both extractants alone were selective toward Er­(III) and Tb­(III), but %E increased significantly, from 86% for Er­(III) and 58% for Tb­(III) with just Cyanex 572 and about 86% for Er­(III) and 33% for Tb­(III) with just Ionquest 801 to 96.8% Er­(III) and 71.7% Tb­(III) when a 50% Ionquest 801 and 50% Cyanex 572 mixture of the extractants was used.^83^ However, separation factors of some light REEs from heavy REEs were higher when Ionquest 801 was used alone, suggesting that adding additional extracting agents is not always worthwhile depending on the goal of the system. Another T-LLE study combined 4-benzoyl-3-phenyl-5-isoxazolone (HPBI) and 2-thenoyltrifluoroacetone (HTTA) with oxime molecules (*meso*-hexamethylpropyleneamine oxime (S2: HM-PAO) and its bis-imine precursor (S1: pre-HM-PAO)) to obtain an acidic/basic ligand mixture.^84^ The authors were able to conclude that HTTA with both oxime molecules exhibited synergistic behavior toward Eu­(III) and that diluent type also had a significant impact.

Hydrophobic ILs have also been employed for synergistic effects. One avenue is adding a small amount to the organic phase of a T-LLE system. Trioctylammonium bis­[(trifluoromethyl)­sulfonyl]­imide ([TOAH]­[Tf_2_N]), a synthesized IL, was added to different organic solvents already containing TODGA for extraction of various REEs from aqueous nitric acid. The effect of the type of diluent containing the IL had a notable effect on the distribution ratio with a mixture of dodecane-octanol (9:1 v/v) having the highest distribution ratio for all lanthanides.^85^

Another option for incorporating an IL as a synergist is dissolving an organic extractant in an undiluted hydrophobic IL making the LP phase primarily an IL (also considered IL-based ATPS, but the aim here is to study the synergistic effects of the ILs and extractants). For example, the commercial extractant N1923 was dissolved in tricaprylmethylammonium nitrate ([A336]­[NO_3_]), an IL easily synthesized from Aliquat 336, for the separation of transition metal ions, vanadium (V­(V)) from chromium (Cr­(VI)).^86^ This system exhibited a synergistic effect for V­(V) while having little effect on Cr­(VI). A very similar system was then applied to REEs where DEHPA was dissolved in the same IL, [A336]­[NO_3_].^81^ Light REEs were synergistically extracted with this method, but heavy REEs demonstrated anti-synergistic behavior which has also been seen in other systems with a mixture of β-diketones.^81,87^

The influence of adding two extractants to an undiluted IL was investigated when a comparison between TODGA in DCE, TODGA in an IL, and TODGA and TBP in an IL and nonane was made. Results showed that the combination of TODGA and TBP in [C_4_MIM]­[Tf_2_N] gave the highest extraction efficiency for all REEs compared to any other combination. Here, a small synergistic effect for light REEs and large effect for the heavy REEs results in high separation factors for pairings of light and heavy REEs: Lu/La, Lu/Sm, and Lu/Tb.^88^ Figure 8a compares the log of the distribution ratio of all Ln­(III) ions plus Y­(III) in the various systems being evaluated. The extraction of all ions was the highest for metal transfer from an aqueous 3 M HNO_3_ phase to the IL phase containing the two ligands TODGA and TBP. The same system had the highest separation factors for the light and heavy metal pairs (Figure 8b).

**Figure 8:**
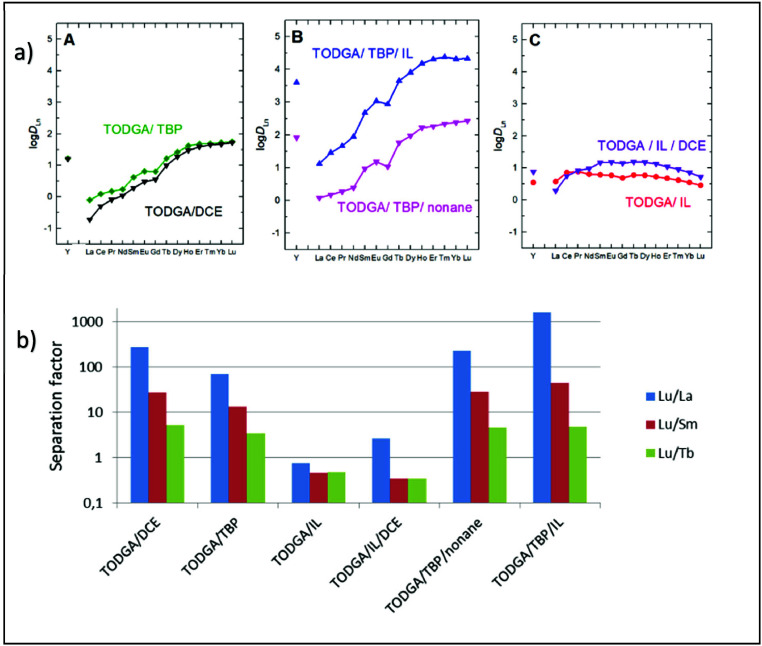
(a) The log of the distribution ratios for all Ln­(III) plus Y­(III) ions and (b) the separation factors for light and heavy REE pairs (Lu/La, Lu/Sm, Lu/Tb) for systems containing various combinations of TODGA, TBP, DCE, nonane, and [C_4_MIM]­[Tf_2_N].^88^ Reproduced or adapted with permission from ref 88. Copyright 2020 Elsevier.

### 4.1 Effects of Auxiliary Agents

Extractants, known as auxiliary agents, have also been added to the aqueous phase in addition to synergistic agents; these are not known as typical synergists as they are water-soluble and exist in the aqueous phase prior to mixing. In 2010, lactic acid (HLac) was added to the aqueous phase of a T-LLE which enhanced extraction efficiency and separation of Pr (III)/Nd (III).^89^ Since then, one or more complexing agents have been added to aqueous phases paired with an organic phase also containing an extractant.^90,91^ It has been found that pairing two complexing agent in the aqueous phase (HLac and citric acid (H_3_cit) increases the overall extraction capacity and separation factors of Ce (III) and Pr (III).^90,91^ Additionally, Yin et al. found that HLac and H_3_cit could be recycled with 10 extraction stages with an alkaline solution.^91^

More recently, a study utilized Cyanex272 dissolved in kerosene mixed with a HCl aqueous phase containing the additional complexing agent, HLac, and different auxiliary agents: citric acid, acetic acid, and Titriplex (III).^92^ They were able to make several conclusions about different combinations of these auxiliary agents in the system: (1) High separation of Pr (III) and Nd (III) from La (III) can be attained without HLac when pH = 5, (2) even small concentrations of HLac increase the %E of REEs, (3) %*E* of REEs is even greater when acetic acid is added as an auxiliary agent, and (4) replacing acetic acid with Titriplex III increased the separation of Pr/Nd compared to when HLac was used alone. It was proposed that the addition of the auxiliary agent acetic acid to HLac created a buffer system to reduce the effect of acidity on the extraction over time.^92^ Cyanex272 in an acidic extractant that extracts REEs by a cation-exchange reaction results in an increase in hydronium ions in the aqueous phase as extraction proceeds. The acetate (Ac^–^) and lactate (Lac^–^) could be combining with these hydronium ions in the aqueous phase. Additionally, acetic acid (p*K*_a_ = 4.76) is a weaker acid than lactic acid (p*K*_a_ = 3.86) and releases less hydronium ions and results in enhanced extraction.^92^ The suggested the use of auxiliary agents can be used in other systems to reduce the effects of acidity of the initial solution and from the extraction mechanism. However, the addition of multiple complexing agents to each phase makes the determination of the extraction mechanism more complex and should be further investigated and reported. Overall, adding auxiliary agents into the aqueous phase has enhanced extraction of selective metal ions in some systems, but the total number of systems published in these systems is still sparse.

## 5 Magnetophoretic Separation

Solvent extraction is the most industrially developed processing route for metal recovery. With many extraction steps and incorporation of other separation techniques such as pyrometallurgical extraction, metal ions can be selectively recovered from ores and waste streams. All separation methods rely on exploiting the differences between some intrinsic properties of the metal ions. The solvent extraction systems are primarily based on variations of ionic radii and metal oxidation states, but the differences between adjacent lanthanides are minimal, making the design of these extraction systems difficult and lengthy. Another intrinsic property, ionic magnetic moment (μ), can also be used for the isolation of selective species. For REEs, the variation of the ionic magnetic moments exceeds that of the ionic radii of the metal ions with coordination numbers of 8 (Table 3). The diamagnetic REEs include those without any unpaired electrons in their valence shell and an ionic magnetic moment of 0 Nm: scandium, yttrium, lanthanum, and lutetium. The remaining REEs have various degrees of paramagnetism.

**Table 3:** Ionic Radii and Ionic Magnetic Moment for All REEs

elemental ions	ionic radii (Å)^93^	ionic magnetic moment (Nm)^94^
La(III)	1.250	0
Ce(III)	1.220	2.6
Pr(III)	1.200	3.6
Nd(III)	1.175	3.7
Pm(III)		
Sm(III)	1.140	1.5
Eu(III)	1.120	3.5
Gd(III)	1.105	7.9
Tb(III)	1.090	9.7
Dy(III)	1.075	10.6
Ho(III)	1.055	10.6
Er(III)	1.040	9.5
Tm(III)	1.025	7.5
Yb(III)	1.010	4.5
Lu(III)	0.995	0
Sc(III)	0.885 ^95^	0
Y(III)	1.019 ^96^	0

Paramagnetic and diamagnetic transition metal ions have been physically separated in an inhomogeneous magnetic field by magnetomigration.^97–99^ It was considered that paramagnetic ions on a silica gel support travel toward the maximum magnetic field as a cluster of metal ions and water molecules rather than individual ions resulting in a large (30 mm) moving distance.^99^ This hypothesis has been employed by others to study the diffusion, magnetomigration, and capture of REEs in a magnetic field. A separation technique termed magnetic field directed crystallization was utilized to separate REE ions of different magnetic susceptibilities and a high SF_La/Dy_ = 494 ± 74 was reported.^1^ By adding a complexing agent to a metal solution consisting of a 1:1 mixture of La/Dy at −25 °C and placing an Fe_14_Nd_2_B permanent magnet outside of the solution, the Dy complex crystallized and migrated toward the magnet.^1,100^ Fan et al. showed that this could also be done at room temperature.^101^ Another magnetic-based system where a quasi-liquid (QL) was formed by adding REEs (Er­(III), Ho­(III), and Y­(III)) to an ionic liquid (IL) that were put under an external magnetic field also showed that diamagnetic and paramagnetic metal ions can be separated.^102^ Drops of QLs were placed on a Petri dish near an external magnetic field. The QL containing Er­(III), the paramagnetic ion, had higher concentration closer to the magnet whereas Y­(III) stayed at the initial location farther from the maximum magnetic field.^102^

Static magnetic fields have also been used in magnetophoretic separation by placing permanent magnets toward the bottom of a cuvette filled with aqueous solutions of the individual metals Ho­(III) and Nd­(III).^103^ The concentration of the solution toward the top of the cuvette was measured over 7 days. While the concentration change of the Nd­(III) solution was unaffected by the magnetic field, Ho­(III), the ion with the larger magnetic moment, experienced a nearly 20% decrease in the concentration in the region away from the magnet suggesting migration to the region with higher field strength.^103^

A similar setup was used to explore the transfer of ions between an organic–aqueous interface.^104^ The magnets were placed at the top of the cuvette near the organic phase to enhance the transfer of ions from the aqueous phase. Unexpectedly, the results of this setup show that there was a decrease in the extraction kinetics of Ho­(III) by the ligand D2EHPA in the magnetic field compared to without a magnetic field.^104^ It was suggested that once the ions are placed in a uniform magnetic field, they interact with both the field and with nearby ions subsequently disturbing the magnetomigration across the interface.^104^ The metal to ligand ratio of the system was 1:1, 0.1 M HoCl_3_ in the aqueous phase, and 0.1 M D2EHPA in the organic phase. Further research might include studying the effect of increasing the metal and ligand concentrations/ratios to explore possible magnetically induced clustering that led to separation in the previously mentioned experiments.^98,99^ In a comparable study by the same group, enhanced magnetic extraction was achieved at higher temperatures (35 °C) until the system without a magnetic field became more efficient after 600 min.^105^

Recent utilization of magnetic separation for transition metal ions is encouraging for applications in REE separation.^106,107^ Rassolov et al. recently developed a numerical model for the diffusion and magnetomigration of metal ions in porous media in a flowing system.^106^ Additionally, a flow system with a separation chamber exhibiting high-gradient magnetic fields generated by stainless steel mesh wool effectively capture approximately 40% of Mn­(II) in an aqueous solution.^107^ These results agree with Fujiwara et al. that paramagnetic metal ions must aggregate in clusters much larger than the size of individual ions.^98,99,107^ In binary mixtures of a paramagnetic and diamagnetic ions, it is likely that the clusters contain both ions decreasing the overall magnetization and, therefore, decreases the capture in the magnetic field.^107^ Lastly, a microfluidic device suppressing forces that impede magnetic separation was utilized for recovery of susceptible ions. However, the trend for the % separations of ions with varying magnetic moments in this system is minimal.^108^

These studies suggest that magnetic separation has the potential to be a significant part of REE separation in the future, and studying the magnetomigration of metal ions and metal complexes in different media will lead to potential strategies and methods for incorporating magnetic separation into REE processing. Combining magnetic and chemical separation methods and utilizing properties of REE ions other than ionic radii will lead to new processing methods that could simplify the current systems. Focusing on the transfer of ions across organic and aqueous interfaces is crucial. Developing a system with green solvents and permanent magnets is ideal for an economically and environmentally friendly approach with less hazardous waste generation and a magnetic field that is reuseable and requires expending little to no energy.

## 6 Technology Readiness Assessment

Technology readiness levels (TRLs) are highly effective in communicating the status of new technologies and system developments. The National Aeronautics and Space Administration (NASA) introduced the 9 TRLs ranging from least technologically mature (e.g., basic technology research) to most mature technology (e.g., systems tests, launch and operations).^109^ For the chemical industry, the TRL ratings are listed in Figure 9a.^110^ The recent modifications to T-LLE summarized in this review all have a TRL < 6 as none of them have been tested in the expected operational environment yet. All ATPS reported here have a TRL 2–3. These systems are still being formulated by investigating the most appropriate components (e.g., concentration of materials, solvent type) for efficient REE extraction at laboratory scales. Similarly, reported solvent extractions with synergistic and auxiliary agents have not reached TRLs higher than 3 as industrial scale set ups were not discussed. Some NAS in this review have been applied to mixer-settler experiments for a somewhat realistic industrial solvent extraction set up resulting in a TRL rating of 5.^66,77^ Magnetophoretic separation technology is still low (TRL 1–2) and researchers are still working to understand magnetomigration and the best conditions for REE separation by cluster formation in a magnetic field. The main challenges in reaching the highest TRLs with these developments are performance, operation cost, consumption of materials, and generation of waste. Figure 9b is a comparison of some of the main industrial concerns regarding separation methods for ATPS, NAS, and systems with synergist and magnetophoretic separation methods to T-LLE (the reference).

**Figure 9:**
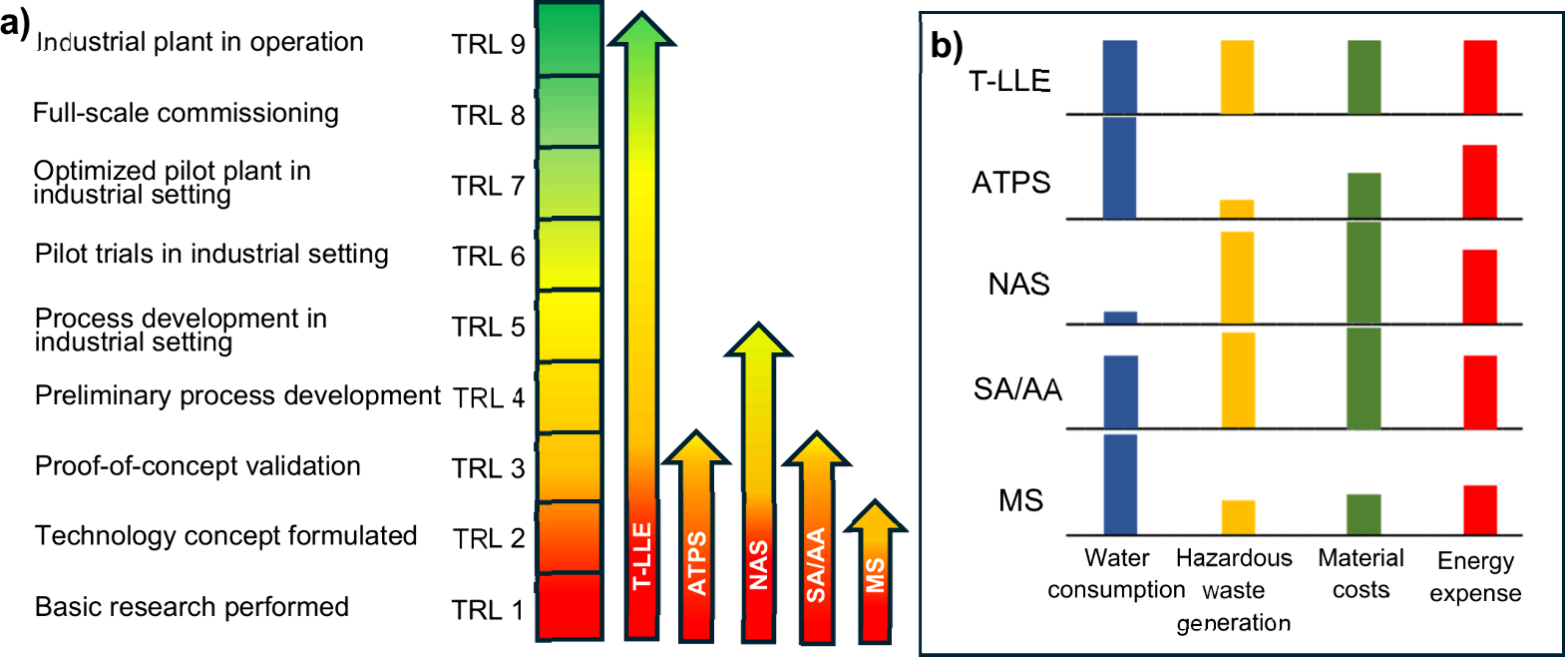
(a) Technology readiness level (TRL) scale105 and the ratings for traditional liquid–liquid extraction (T-LLE), aqueous two-phase systems (ATPS), nonaqueous systems (NAS), addition of synergist agents/auxiliary agents (SA/AA) and magnetophoretic separation (MS). (b) An estimated comparison of the water consumption, hazardous waste generation, material costs, and energy expense for T-LLE, ATPS, NAS, SA/AA, and MS.

## 7 Conclusion

The processing of REEs is a challenging task due to the complicated chemistry and engineering involved in designing a system aimed at separating elements with similar properties. Geopolitical competition and minimal mining and processing operations in most countries have resulted in focusing on REE production and efficient separation procedures from primary sources and waste streams where REEs can be recycled. Being that solvent extraction is the most common laboratory and industrial REE separation method, this review summarizes recent modifications to the traditional liquid–liquid extraction (T-LLE) system. The development of aqueous two-phase systems (ATPS) gives rise to systems avoiding hazardous solvents and the generation of toxic waste. However, finding optimal conditions for the ternary system to become a two-phase system and have efficient extraction is a challenge and, if applied to industrial scale REE processing, would generate an abundant amount of contaminated wastewater, the disposal of which is already a large percentage of the REE production cost. A limited number of nonaqueous systems (NAS) have been developed for REEs, but large-scale NAS seem achievable as efficient extraction resulted from mixer-settler experiments with green nonaqueous solvents such as DES. Adding synergistic agents to all system types (T-LLE, ATPS, and NAS) is a promising route for enhancing existing systems. When one or more metals experience an anti-synergistic effect and selective metals experience synergism, separation factors will be enhanced. Lastly, utilizing the differences in ionic magnetic moments of REEs in addition to their differences in ionic radii and oxidation states could enhance their overall extraction efficiencies and lead to shorter refinement processes, saving time, product, consumption, and waste generation. The behavior of metal ions in an applied magnetic field for separation needs to be explored further to design systems worth applying to industry for the goal of increasing the supply of REEs.

## Abbreviations

%*E*: percent extraction
μ: ionic magnetic moment
ABS: aqueous biphasic systems
ATPS: aqueous two-phase systems
*D*_A_: distribution ratio
DES: deep eutectic solvent
*D*_M_: distribution ratio of all molecular conditions
ECE: energy critical element
H: relative distribution enhancement factor
IL: ionic liquid
*K*_d_: distribution coefficient
LP phase: less polar phase
MP phase: more polar phase
MS: magnetophoretic separation
NAS: nonaqueous systems
*P*: partition coefficient
PPIL: pseudoprotic ionic liquid
*R*: synergistic enhancement coefficient
REE: rare earth element
REO: rare earth oxide
SA/AA: synergistic agents/auxiliary agents
SC: synergistic enhancement coefficient
SF_*A*/*B*_: separation factor
T-LLE: traditional liquid–liquid extraction
TREO: total rare earth oxide
TRL: technology readiness level
